# The sTREM2 Concentrations in the Blood: A Marker of Neurodegeneration?

**DOI:** 10.3389/fmolb.2020.627931

**Published:** 2021-03-09

**Authors:** Evelyn Ferri, Paolo Dionigi Rossi, Annalisa Geraci, Simona Ciccone, Matteo Cesari, Beatrice Arosio

**Affiliations:** ^1^Geriatric Unit, Fondazione IRCCS Ca' Granda Ospedale Maggiore Policlinico, Milan, Italy; ^2^Department of Clinical Sciences and Community Health, University of Milan, Milan, Italy; ^3^Geriatric Unit, IRCCS Istituti Clinici Scientifici Maugeri, Milan, Italy

**Keywords:** TREM2, Alzheimer's disease, idiopathic normal pressure hydrocephalus, neurodegeneration, biomarker

## Abstract

Microglia performs a variety of functions during brain development designed to maintain brain homeostasis. Triggering receptor expressed on myeloid cells 2 (TREM2) is expressed in microglial cells modulating phagocytosis, cytokine production, cell proliferation, and cell survival. Interestingly, the levels of soluble TREM2 (the secreted ectodomain of TREM2, sTREM2) were higher in cerebrospinal fluid (CSF) from Alzheimer's disease (AD) patients than subjects without cognitive decline. It is noteworthy that, while CSF sTREM2 levels have been extensively studied, few studies have investigated sTREM2 in blood producing conflicting results. We aimed to investigate the levels of sTREM2 in CSF and blood from a cohort of well-characterized AD comparing the results to those obtained in patients suffering from idiopathic normal pressure hydrocephalus (iNPH), a potentially reversible cognitive impairment. Our findings underlined a significantly lower plasma sTREM2 concentration in AD patients compared to iNPH subjects [39.1 ng/mL (standard deviation (SD), 15.0) and 47.2 ng/mL (SD, 19.5), respectively; *p* = 0.01], whereas no difference was revealed between the two groups in the CSF sTREM2 levels. The adjusted regression analyses evidenced in AD patients an association between plasma and CSF sTREM2 levels [*B* = 0.411; 95% confidence interval (CI), 0.137–0.685, *p* = 0.004], as well as β-amyloid concentrations (*B* = 0.035; 95% CI, 0.007–0.063, *p* = 0.01) and an association between CSF sTREM2 and phospho-Tau concentrations (*B* = 0.248; 95% CI, 0.053–0.443; *p* = 0.01). No significant relation was found in iNPH patients. In conclusion, these differences in sTREM2 profiles between AD and iNPH reinforce the notion that this receptor has a role in neurodegeneration.

## Introduction

Microglial cells are the resident immune cellular population of the central nervous system (CNS) (Deczkowska et al., 2018). Current evidence indicates that microglia performs a variety of functions during brain development designed to maintain brain homeostasis. Indeed, thanks to a set of sensory mechanisms, microglia can modulate physiological programmed cell death (Vaux and Korsmeyer, 1999), phagocytosis of dying cells and cell debris (Lampron et al., 2013), synaptic pruning (Zhang et al., 2014; Lui et al., 2016), sculpted synaptic connectivity (Bialas and Stevens, 2013; Miyamoto et al., 2016), and vascular branching (Ransohoff and El Khoury, 2015).

The deterioration of the microglia cell during aging might be crucial for the development of specific age-related diseases. For example, in Alzheimer's disease (AD), neuroinflammation exerts a key role in the disease progression. It has been demonstrated that β-amyloid (Aβ) can activate microglia cells and initiate the chronic inflammatory response (at least, partially) responsible for neurodegeneration (Mosher and Wyss-Coray, 2014; Heneka et al., 2015; Ransohoff, 2016). Although microglia activation and involvement in AD pathogenesis have been established, its function is still undefined as microglia can also phagocytize and degrade Aβ and produce anti-inflammatory cytokines to counterbalance inflammatory response (Anwar and Rivest, 2020).

Interestingly, Genome-Wide Studies show that approximately two-thirds of new AD-risk single-nucleotide polymorphisms are exclusively or most highly expressed in microglia (Jendresen et al., 2017). Among these risk genes, triggering receptor expressed on myeloid cells 2 (TREM2) is associated with the highest risk of developing AD, increasing the risk by 2- to 4-fold (Gratuze et al., 2018).

The impaired signaling of TREM2 has been associated with abnormalities in phagocytosis, cytokine production, cell proliferation, and cell survival. In particular, during aging, microglial cells become hyperresponsive (primed) to inflammation, leading to an increased release of proinflammatory cytokines. In other words, the progressive decline of brain TREM2 expression can contribute to switching the microglia phenotype into a neurodegenerative pattern (Mecca et al., 2018).

Moreover, it has been reported that the lack of TREM2 leads to increased Aβ burden (Wang et al., 2015; Zhao, 2019) and decreased degradation (Raha-Chowdhury et al., 2015), as well as up-regulation of proinflammatory gene expressions (e.g., tumor necrosis factor α and nitric oxide synthase 2) (Takahashi et al., 2005). Instead, the overexpression of TREM2 helps the maintenance of microglial homeostasis and induces the decrement of Aβ deposition (Takahashi et al., 2005; Jiang et al., 2014; Lee et al., 2018).

Interestingly, soluble TREM2 (the secreted ectodomain of TREM2, sTREM2) has been found in cerebrospinal fluid (CSF) from AD patients, where it exerts a proinflammatory action (Zhong et al., 2017), in a higher level when compared to controls (Shen et al., 2019).

Recently, Ma et al. (2020) have reported that sTREM2 levels change temporally with AD progression, evidencing that low CSF Aβ is associated with a decrease in CSF sTREM2 levels, whereas increased sTREM2 is associated with increased Tau levels.

Detectable expression of TREM2 was described in peripheral blood mononuclear cells from mild cognitive-impaired patients that later converted to AD (Casati et al., 2018), as well as in blood from subjects with an increased risk to develop dementia in a Japanese cohort (Ohara et al., 2019). It is noteworthy that, while CSF sTREM2 levels have been extensively studied, only a few studies have investigated sTREM2 in blood, often producing conflicting results (Piccio et al., 2008; Kleinberger et al., 2014; Ashton et al., 2019).

Under these premises, we aimed to (i) study the concentrations of sTREM2 in CSF and blood from a cohort of well-characterized persons with AD, (ii) correlate the concentrations with CSF markers of AD progression, and (iii) compare the results shown in the AD group to those obtained in a cohort of patients suffering from a potentially reversible cognitive impairment [i.e., due to idiopathic normal pressure hydrocephalus (iNPH)] (Ott et al., 2010; Di Ieva et al., 2014).

## Materials and Methods

### Study Design

The study involved 76 well-characterized AD patients and 45 iNPH subjects with biobanked plasma and biobanked CSF (37 AD and 42 iNPH). Thirty-three individuals without cognitive decline (CT), age-, and sex-matched with the AD group, were selected to compare the plasmatic concentrations of sTREM2.

The diagnosis of AD was made according to the criteria proposed by Dubois et al. (2014). In contrast, the iNPH diagnosis was formulated according to International Guidelines published in 2005 (Marmarou et al., 2005). As all the selected iNPH subjects were ineligible for the surgical procedure, the CSF from iNPH patients was obtained by a “tap test” procedure, as previously described (Rossi et al., 2019).

The study protocol received approval from the local ethical committee. All subjects gave their informed consent to participate in the study.

### Biochemical and Molecular Determinations

The CSF concentrations of Aβ_1−42_, Tau, and phospho-Tau (pTau) were assessed using the commercially available enzyme-linked immunosorbent assay (ELISA) kits (Innogenetics, Ghent, Belgium). The CSF and plasma concentrations of sTREM2 were measured using the Human TREM2 ELISA kit (Abcam, Cambridge, MA, USA) according to the manufacturer's instructions. The apolipoprotein E (APOE) genotype was determined as previously described (Ferri et al., 2019).

### Statistical Analysis

SPSS statistical package was used to conduct the statistical analyses (SPSS version 26, Chicago, IL, USA). All the variables were normally distributed. Results are expressed as mean [standard deviation (SD)] and/or percentage. Student's *t*-test was used for the comparisons between groups. The χ^2^ Pearson model was used to calculate sex and APOE ε4 allele distribution. Spearman's correlation coefficients were calculated to evaluate the relationship between all variables. Linear regression analysis was used to test the association between sTREM2 concentrations and the other variables independently of multiple confounding factors (i.e., sex, age, and presence of APOE ε4 allele). A *p* < 0.05 was considered as the threshold for statistical significance.

## Results

Table 1 shows the characteristics of the subjects. As described in Table 1, iNPH patients were significantly older than AD patients and CT subjects. The percentages of women were higher in AD than iNPH, whereas those of ApoE ε4 carriers were higher in AD than iNPH and CT subjects. Mini-Mental State Examination score did not differ between AD and iNPH and was suggestive of cognitive impairment in both groups.

**Table 1:** Characteristics of study participants.

	**AD**	**iNPH**	**CT**
Age	78.1 (4.6)	83.7 (5.5)^a^	78.7 (5.6)
Sex (% women)	69.7%^b^	46.8%	60.6%
ApoE (% ε4 carriers)	46.1%^c^	12.8%	6.3%
MMSE	22.1 (4.6)	23.9 (5.1)	28.9 (1.1)^d^
Aβ_1−42_ (pg/mL)	486.5 (136.8)^b^	748.8 (312.3)	n.d.
Tau (pg/mL)	721.9 (524.9)^b^	173.3 (101.8)	n.d.
pTau (pg/mL)	89.6 (42.6)^b^	34.2 (13.5)	n.d.
Plasma sTREM2 (ng/mL)	39.1 (15.0)^b^	47.2 (19.5)	44.4 (15.1)
CSF sTREM2 (ng/mL)	32.6 (18.1)	36.4 (13.2)	n.d.

*Data are expressed as mean [standard deviation (SD)] and percentages*.

*ApoE, Apolipoprotein E; MMSE, mini-mental state examination; Aβ_1-42_, β-amyloid 1–42; pTau, phospho-Tau; CSF, cerebrospinal fluid; n.d., not detected*.

a *p < 0.001 vs. AD and CT*.

b *p < 0.01 vs. iNPH*.

c *p < 0.001 vs. iNPH and CT*.

d *p < 0.001 vs. AD and iNPH*.

The AD patients showed significantly different CSF biomarkers concentrations compared to iNPH subjects (Table 1). Interestingly, we observed significantly lower plasma sTREM2 concentrations in AD patients compared to iNPH subjects, whereas no difference was revealed between the two groups in the CSF sTREM2 levels (Table 1). Although statistical significance was not achieved, the group of CT had plasmatic concentrations of sTREM2 higher compared to AD patients (*p* = 0.09) (Table 1).

AD patients showed negative correlations of age with CSF Tau and pTau, whereas a positive correlation was reported between age and plasma sTREM2 concentrations (Table 2). In the iNPH group, age only correlated with the CSF sTREM2, whereas in the CT subjects, age slightly correlated with the sTREM2 plasma concentrations (Table 2).

**Table 2:** Correlation between age and Aβ_1−42_, Tau, pTau, plasma sTREM2, and CSF sTREM2 concentrations according to the three study groups.

**Age**	**AD**		**iNPH**		**CT**	
	**ρ**	***p*-value**	**ρ**	***p*-value**	**ρ**	***p*-value**
Aβ_1−42_ (pg/mL)	0.053	0.70	−0.186	0.30	n.d.	n.d.
Tau (pg/mL)	−0.336	**0.01**	0.201	0.26	n.d.	n.d.
pTau (pg/mL)	−0.267	**0.05**	0.226	0.21	n.d.	n.d.
Plasma sTREM2 (ng/mL)	0.322	**0.005**	0.053	0.74	0.349	**0.05**
CSF sTREM2 (ng/mL)	0.156	0.35	0.400	**0.01**	n.d.	n.d.

*Bold values represent significance level p < 0.05. ρ, Spearman's correlation coefficient; Aβ_1-42_, β-amyloid 1–42; pTau, phospho-Tau; CSF, cerebrospinal fluid; n.d., not detected*.

Regarding biomarkers, in AD patients, a positive correlation was found between plasma and both CSF sTREM2 and Aβ_1−42_ concentrations, whereas no significant correlation was highlighted in iNPH subjects (Table 3). Furthermore, in the AD group, CSF sTREM2 positively correlated with Aβ_1−42_, Tau, and pTau concentrations (Table 3). Instead, in the iNPH group, only a positive correlation between CSF sTREM2 and pTau was observed (Table 3).

**Table 3:** Correlation between plasma sTREM2 and Aβ_1−42_, Tau, pTau, and CSF sTREM2 concentrations (A); and between CSF sTREM2 and Aβ_1−42_, Tau, pTau, and plasma sTREM2 concentrations (B) in AD and iNPH subjects.

	**AD**		**iNPH**	
	**ρ**	***p-*value**	**ρ**	***p-*value**
**(A) Plasma sTREM2**				
Aβ_1−42_ (pg/mL)	0.332	**0.01**	0.039	0.83
Tau (pg/mL)	0.085	0.53	0.232	0.19
pTau (pg/mL)	0.199	0.14	0.127	0.48
CSF sTREM2 (ng/mL)	0.476	**0.003**	0.216	0.19
**(B) CSF sTREM2**				
Aβ_1−42_ (pg/mL)	0.332	**0.04**	0.128	0.48
Tau (pg/mL)	0.343	**0.04**	0.137	0.45
pTau (pg/mL)	0.377	**0.02**	0.354	**0.04**
Plasma sTREM2 (ng/mL)	0.476	**0.003**	0.216	0.19

*Bold values represent significance level p < 0.05. ρ, Spearman's correlation coefficient; Aβ_1-42_, β-amyloid 1–42; pTau, phospho-Tau; CSF, cerebrospinal fluid*.

The regression analyses adjusted by age, sex, and presence of the ε4 allele of the ApoE gene confirmed in AD patients the association between plasma sTREM2 levels and CSF levels (*B* = 0.411; 95% CI, 0.137–0.685; *p* = 0.004), as well as Aβ_1−42_ concentrations (*B* = 0.035; 95% CI, 0.007–0.063; *p* = 0.01). Moreover, regression analysis highlighted an association between CSF sTREM2 and pTau concentrations (*B* = 0.248; 95% CI, 0.053–0.443; *p* = 0.01). No significant relation was found in iNPH patients.

## Discussion

This study provides several interesting findings. First, lower plasmatic concentrations of sTREM2 were found in our well-characterized AD patients compared to both iNPH and controls without cognitive decline. Interestingly, in our AD group, there was a significant association between CSF sTREM2 and plasma sTREM2 levels, a result that was not found in iNPH subjects. Moreover, in AD patients, we found a positive association between CSF sTREM2 and pTau concentrations (consistently with previous findings) (Ma et al., 2020) and between plasmatic levels of sTREM2 and CSF Aβ_1−42_ concentrations (differently from previous reports) (Bekris et al., 2018).

TREM2 has a role in protecting against neuroinflammation by the inhibition of the persistent activation of microglia, promoting phagocytosis, and clearing apoptotic neurons (Colonna and Wang, 2016). This receptor is expressed on cells of the myeloid lineage, including microglia, dendritic cells, granulocytes, bone marrow, monocyte-derived macrophages, and tissue macrophages (Jay et al., 2017).

An exciting aspect of TREM2 resides in releasing its soluble variant in various human biofluids, such as CSF and blood (Bekris et al., 2018). It has been speculated that changes in the sTREM2 concentration in these biofluids might be a potential clue attributed to microglial dysfunction and neuroinflammation typical of AD (Liu et al., 2018).

Existing studies report no significant difference in the plasma levels of sTREM2 between AD cases and healthy controls (Piccio et al., 2016; Liu et al., 2018). On the other hand, our group has previously demonstrated that in peripheral blood mononuclear cells, TREM2 seems to play a protective role in the preclinical stage of AD (Casati et al., 2018).

Evidence suggests that sTREM2 is able to prevent apoptosis in macrophages (Wu et al., 2015) and to promote cell survival (Wu et al., 2015), proliferation, and migration (Zhong and Chen, 2019). Even if not directly to AD, the pathways involved in TREM2 protective role have been better dissected in cancer models. In particular, TREM2 blockade has been shown to significantly promote the proliferation of colorectal cancer cells by regulating cell cycle–related factors such as p53, p21, and cyclin D1 (Kim et al., 2019). Interestingly, peripheral blood mononuclear cells from AD patients express an abnormal and detectable conformational state of p53. This anomaly allows distinguishing these cells from those of cognitively healthy subjects with the same age (Lanni et al., 2008). This is particularly intriguing in light of the consideration that appears to be an inverse correlation between cancer and AD due to shared biological mechanisms (Lanni et al., 2020).

Under these premises, in this study, we compared two neurological diseases, both characterized by cognitive impairment and difficult to differentiate from each other without diagnostic insights (Jingami et al., 2015). It is to note that iNPH is characterized by neurological symptoms that occur as a result of the impairment of CSF clearance and its accumulation in the brain (Ott et al., 2010; Di Ieva et al., 2014). In many cases, these symptoms revert after the drainage of the CSF (Rossi et al., 2019).

The results of our study highlighted lower plasmatic levels of sTREM2 in patients with AD compared to those with iNPH. Interestingly, we did not find substantial differences comparing the sTREM2 plasma levels between iNPH and control subjects characterized by normal cognitive functions. These results suggest that plasmatic sTREM2 may be a useful marker to identify cognitive impairment due to neurodegenerative processes. Moreover, the comparable results obtained in iNPH and CT subjects confirmed our previous findings showing blood and CSF protein profiles similar in these two groups (Fania et al., 2017; Torretta et al., 2018).

Regarding CSF, our results did not show a difference in sTREM2 concentrations between overt AD and iNPH patients, as already reported in studies that compare AD and healthy controls (Henjum et al., 2016; Banerjee et al., 2020). This finding confirmed recent studies demonstrating that the CSF concentrations of sTREM2 increase in a disease stage–dependent manner. Indeed, CSF sTREM2 levels seem to reach a peak in the early symptomatic phase of AD and then decrease when the disease becomes overt (Suarez-Calvet et al., 2016; Ma et al., 2020).

Moreover, our study results confirmed the positive association between CSF sTREM2 and plasma sTREM2 in AD patients (Bekris et al., 2018). This association disappeared in the iNPH group. In the study of Bekris et al. (2018), CSF sTREM2 levels also correlated with a marker of blood–brain barrier disruption (CSF albumin–to–serum albumin ratio), raising the possibility that this latter may account for the relationship observed between peripheral and central sTREM2 concentrations in AD (Bekris et al., 2018).

The regression analyses highlighted an association between CSF sTREM2 with pTau, but not with Aβ_1−42_ concentrations in AD patients, suggesting that sTREM2 might play a role in the pathological processes occurring after Aβ_1−42_ accumulation (Zhong and Chen, 2019). These results further support the hypothesis that changes in CSF sTREM2 may be associated with neuronal injury characterizing neurodegeneration (Henjum et al., 2016; Heslegrave et al., 2016; Piccio et al., 2016; Suarez-Calvet et al., 2016; Gispert et al., 2017; Zhong and Chen, 2019).

Interestingly, despite the fact that a recent study stated that plasma sTREM2 did not significantly correlate with CSF AD-related biomarkers (Bekris et al., 2018), we found a positive association between plasma sTREM2 and the CSF concentrations of Aβ_1−42_ in AD. Although the function of TREM2 in AD pathology is partly unclear, it is hypothesized a role of TREM2 in clearing the soluble Aβ aggregates and other toxic debris and controlling the inflammatory reactions elicited by the AD pathology (Hsieh et al., 2009; N'Diaye et al., 2009; Kleinberger et al., 2014). Assuming the progressive disruption of the blood–brain barrier occurring in AD (Bekris et al., 2018), sTREM2 engaged in counteracting Aβ-deposits in the CNS could cross the blood–brain barrier to the bloodstream, justifying the positive correlation observed in AD between plasma sTREM2 and Aβ_1−42_ CSF concentrations.

However, we could not exclude the fact that aging, the most significant risk factor for AD, may be in part responsible for the sTREM2 alterations that emerged from our results. Indeed, aging is associated with gliosis, increased microglial activity (Heneka et al., 2014), and astrocytosis in the brain (Nichols et al., 1993), which have been proven to increase sTREM2 concentrations to contrast these altered processes (Liu et al., 2018). In this regard, a previous study showed a threefold enrichment of CSF sTREM2 concentrations from 50 to 90 years old (Forabosco et al., 2013; Henjum et al., 2016). This may explain the association between age and CSF sTREM2 concentrations observed in iNPH subjects significantly older than the other two groups.

In our cohort of subjects, plasma sTREM2 concentrations strongly correlated with age in AD patients and slightly in the CT group, whereas there was no correlation in iNPH subjects. Despite these assumptions, the association between CSF sTREM2 and plasma sTREM2 levels was confirmed in the AD group even after adjusting for age and sex, further supporting a role of TREM2 in the pathobiology of AD.

There are limitations to this study. First, the number of the study participants is limited, and thus these findings are preliminary and need to be further investigated in large cohorts of patients. Another limitation is the CSF unavailability for subjects without cognitive decline. Indeed, we were not able to collect CSF from these subjects for ethical reasons, contrarily to AD and iNPH subjects for which CSF has been collected for diagnostic purposes (Dubois et al., 2014; Rossi et al., 2019).

In conclusion, we demonstrate that plasma levels of sTREM2 are decreased in AD patients, but not in iNPH and subjects without cognitive decline. Moreover, only in the AD group, we observe a significant association between CSF sTREM2 and plasma sTREM2 levels, not evidenced in iNPH subjects. These significant differences in sTREM2 profiles between AD and iNPH reinforce the notion that this receptor has a role in neurodegeneration.

## Data Availability Statement

The raw data supporting the conclusions of this article will be made available by the authors, without undue reservation.

## Ethics Statement

The studies involving human participants were reviewed and approved by Fondazione IRCCS Cà Granda Ospedale Maggiore Policlinico. The patients/participants provided their written informed consent to participate in this study.

## Author Contributions

BA conceived and designed the study. BA and EF analyzed the data, interpreted the data, and results. BA, EF, and MC drafted the manuscript. MC provided critical revision. PDR and SC performed clinical assessment. AG performed biochemical analyses. All authors contributed to the article and approved the submitted version.

## Conflict of Interest

The authors declare that the research was conducted in the absence of any commercial or financial relationships that could be construed as a potential conflict of interest.
